# Hemichorea Hemiballism Syndrome: The First Presentation of Type 2 Diabetes Mellitus as a Rare Cause of Chorea

**Published:** 2011-03-30

**Authors:** P. Mittal

**Affiliations:** 1 Assistant Professor, Department of Radiodiagnosis, Punjab Institute of Medical Sciences, Jalandhar, India

**Keywords:** Hemichorea, Hemiballism, MRI, Ischemia, Diabetes

## Abstract

Hemichorea-hemiballism (HCHB) syndrome, which is most commonly related to non-ketotic hyperglycemia, is a rare type of chorea. Here, we present an unusual case of HCHB syndrome who was not a known case of diabetes. This case highlights the importance of recognising underlying non-ketotic hyperglycemia, as control of hyperglycemia is helpful in the quick relief of symptoms.

## Introduction

The most common cause of hemichorea hemiballism (HCHB) syndrome is the vascular insult in contralateral striatum or subthalamic nucleus, however it can occur secondary to a variety of causes like tumors, encephalitis, neurodegenerative disorders, drugs, ipsilateral chronic subdural hemorrhage and metabolic disorders such as non-ketotic hyperglycemia.[1][2] HCHB syndrome is the most common cause of unilateral chorea in type 2 diabetes mellitus patients with the underlying mechanism of non-ketotic hyperglycemia. The characteristic imaging manifestation is T1 shortening in the contralateral corpus striatum.[1] Rarely, HCHB syndrome may be the first presentation of type 2 diabetes; therefore, it should always be included in the differential diagnosis of chorea, especially in elderly patients.[3]

## Case Presentation

**Fig. 1:**
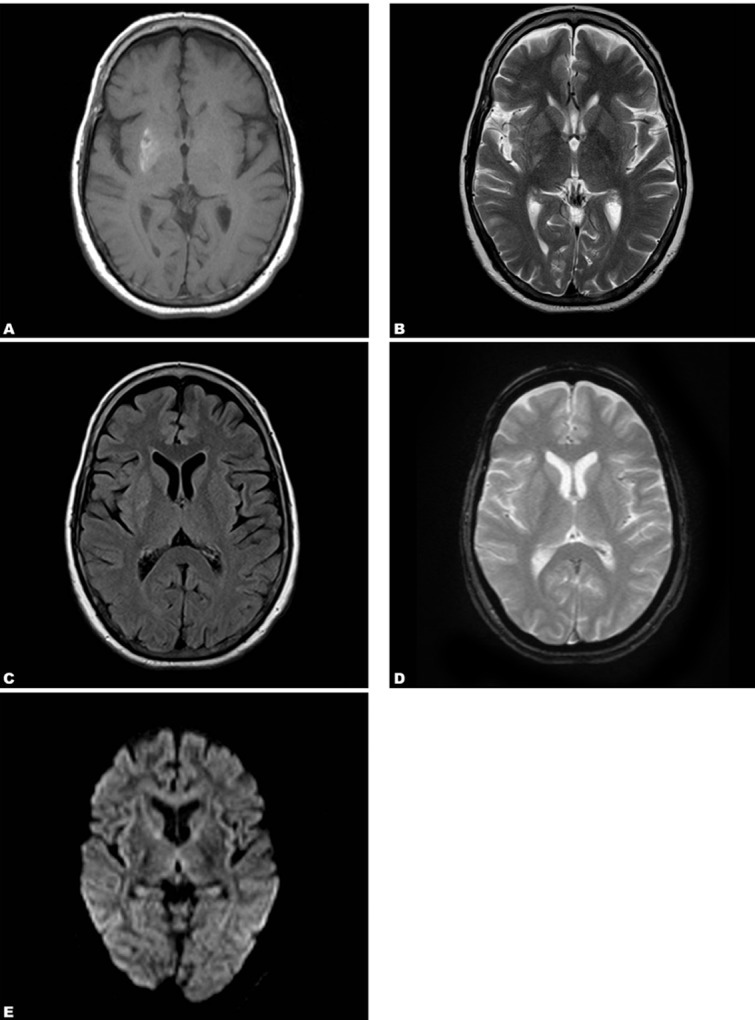
A 60-year-old female patient with HCHB syndrome. (A. Hyperintense signal is seen in the right caudate nucleus and putamen on T1W image. B & C. Subtle hyperintense signal is seen in the caudate nucleus and putamen on T2W and FLAIR images. D & E. No signal abnormality is seen on GRE and DW images.)

A 60-year-old female patient, a known case of ischemic heart disease, presented with complaints of acute onset quasi-purposive slow writhing movements mainly involving the distal left upper limb and lower limb from four hours prior to admission. There was no history of trauma, diabetes mellitus, hypertension or stroke and also no family history of movement disorders. On examination, she was afebrile, normotensive, fully conscious and oriented. No weakness was seen on any side. Magnetic resonance imaging (MRI) was performed using a dedicated 1.5T scanner. T1-weighted images showed hyperintensity in the right lentiform nucleus (Fig. 1A). Subtle hyperintense signal was seen on T2W/FLAIR images (Figs. 1. B & C). No evidence of blooming or diffusion restriction was seen (Figs. 1. D & E). The possibility of hyperglycemia-induced HCHB syndrome was kept in mind and the blood glucose levels were advised. Fasting blood glucose level was elevated (346 mg/ dl) and no ketones were detected in urinalysis. This confirmed the diagnosis of type 2 diabetes mellitus with non-ketotic hyperglycemia. She was started on oral hypoglycemics and glycemic control was achieved. Receiving haloperidol and valproate, the patient improved dramatically. The symptoms disappeared completely within two days and she was discharged in stable condition.

## Discussion

HCHB syndrome presents with unilateral choreoathetoid movements of both upper and lower limbs. Non-ketotic hyperglycemia is an unusual cause of HCHB syndrome. In a series of 51 patients with sporadic chorea, non-ketotoic hyperglycemia was only responsible for two of the patients.[4] Although HCHB syndrome is the most common movement disorder in non-ketotic hyperglycemia, it may also be associated with other rare disorders like orobucco-lingual dyskinesia.[1] The characteristic imaging finding of HCHB syndrome is T1 hyperintensity in contralateral corpus striatum. An abnormal signal may extend into the globus pallidus and up to the medial part of the cerebral peduncle in the midbrain along the striatonigral pathway. Neurons in the pathway are believed to play a critical role in the genesis of abnormal movements.[5] Patchy involvement of the caudate nucleus and putamen seems to be prerequisite for the development of HCHB syndrome. It has also been demonstrated in animal studies that it is the partial rather than complete lesions of the caudate and putamen which result in contralateral abnormal movements.[5] Although imaging findings are characteristic, the origin of T1 hyperintensity remains speculative. There are many theories like ischemia, metabolic acidosis, petechial hemorrhages, depletion of acetyl choline or abundant gemistocytes,[3][5] among which ischemia is considered to be the most plausible explanation. In a study, reduced metabolic activity has been demonstrated in the affected region using positron emission tomography (PET) imaging, which could be due to ischemic insult.[6] Because of the reversible nature of the condition, ischemic injury is more likely partial and reversible, but the selective vulnerability of basal ganglia is not explained. The striatal hyperintensity may diminish or persist for months or years after resolution of the movement disorder.[3]

The differential diagnosis of T1 hyperintense lesion in the basal ganglia is wide, including methemoglobin in intracranial hemorrhage, manganese deposition due to parenteral nutrition, copper accumulation in Wilson’s disease, hemorrhagic infarction, Japanese encephalitis, calcification, hamartoma (neurofibromatosis-1), hypoxic ischemic encephalopathy, acquired hepatocerebral degeneration and HCHB syndrome.[7] All the systemic causes are easily excluded in our case because they are usually bilateral. Hemorrhagic infarction is unlikely because there was no diffusion restriction of the lesion and no blooming was detected on gradient echo images. Although Japanese encephalitis can be initially unilateral, near normal T2W and FLAIR images and its non-supportive history exclude this diagnosis. Calcification is also usually bilateral and will not be present with HCHB syndrome. Moreover, imaging findings did not support any tumor or vascular malformation as no mass effect and no vascular flow voids were seen. These observations along with characteristic imaging findings led us to the diagnosis of non-ketotic hyperglycemia-induced HCHB syndrome.

Chorea is a relatively uncommon neurological manifestation of diabetes mellitus. HCHB is most commonly related to non-ketotic hyperglycemia in diabetic patients. It is usually seen in elderly patients with female preponderance.[7] The majority of cases are reported to be Asian females, which could be related to an underlying genetic predisposition.[8] There have been isolated case reports of HCHB presenting as the first manifestation of type 2 diabetes mellitus.[1][3][9][10][11] Most of these cases are elderly females with no known previous history of diabetes mellitus.[10] In these cases, it is important to recognize the underlying cause because correction of the underlying hyperglycemia usually leads to quick relief of the symptoms.
